# Proteomic analysis of the periodontal pathogen *Prevotella intermedia* secretomes in biofilm and planktonic lifestyles

**DOI:** 10.1038/s41598-022-09085-0

**Published:** 2022-04-04

**Authors:** Maribasappa Karched, Radhika G. Bhardwaj, Muawia Qudeimat, Areej Al-Khabbaz, Arjuna Ellepola

**Affiliations:** 1 Oral Microbiology Research Laboratory, Department of Bioclinical Sciences, Faculty of Dentistry, Health Sciences Center, Kuwait University, PO Box 24923, 13110 Safat, Kuwait; 2 Department of Developmental and Preventive Sciences, Faculty of Dentistry, Health Sciences Center, Kuwait University, Safat, Kuwait; 3 Department of Surgical Sciences, Faculty of Dentistry, Health Sciences Center, Kuwait University, Safat, Kuwait

**Keywords:** Cellular microbiology, Biofilms

## Abstract

*Prevotella intermedia* is an important species associated with periodontitis. Despite the remarkable clinical significance, little is known about the molecular basis for its virulence. The aim of this study was to characterize the secretome of *P. intermedia* in biofilm and planktonic life mode. The biofilm secretome showed 109 proteins while the planktonic secretome showed 136 proteins. The biofilm and the planktonic secretomes contained 17 and 33 signal-peptide bearing proteins, 13 and 18 lipoproteins, respectively. Superoxide reductase, sensor histidine kinase, C40 family peptidase, elongation factor Tu, threonine synthase etc. were unique to biofilm. Of the ~ 30 proteins with predicted virulence potential from biofilm and planktonic secretomes, only 6 were common between the two groups, implying large differences between biofilm and planktonic modes of *P. intermedia*. From Gene Ontology biofilm secretome displayed a markedly higher percent proteins compared to planktonic secretome in terms of cellular amino acid metabolic process, nitrogen compound metabolic process etc. Inflammatory cytokine profile analysis revealed that only the biofilm secretome, not the planktonic one, induced important cytokines such as MIP-1α/MIP-1β, IL-1β, and IL-8. In conclusion, the revealed differences in the protein profiles of *P. intermedia* biofilm and planktonic secretomes may trigger further questions about molecular mechanisms how this species exerts its virulence potential in the oral cavity.

## Introduction

Bacteria release proteins and other components into extracellular milieu continuously during normal growth and physiology. Many of the secreted proteins often function in nutrient acquisition, but in pathogenic bacteria, they play a key role in disease, e.g., by helping the bacteria in host colonization or by modulating host immune responses^1,2^. Thus, extracellular secretion of bacterial proteins is an important virulence mechanism. To achieve this, bacteria have devised various strategies, e.g., dedicated secretory systems^3,4^ and extracellular vesicles^5,6^. The central components of the main protein translocation system, the Sec system, share a high degree of sequence similarity between Gram-positive and Gram-negative bacteria.

Oral infectious diseases such as caries and periodontitis are dental plaque biofilm-driven^7,8^. Dental plaque is a structurally and functionally organized, highly complex multispecies biofilm. The resident bacteria in this multispecies community exhibit extensive interactions while forming biofilm structures, carrying out physiological functions, and inducing microbial pathogenesis^9^. Interspecies interactions in biofilms are competitive, cooperative and, antagonistic^10^. To facilitate such interactions, plaque bacteria may release several cellular components into the extracellular space within the biofilm matrix. In addition, proteins secreted in biofilms may have specific effects in terms of the virulence properties of biofilm residents, which could influence the overall pathogenicity of biofilms.

Periodontitis is a chronic inflammatory disease characterized by mild to moderately severe inflammation of the periodontal tissue, progressive destruction of ligament fibers, as well as alveolar bone loss^11^. The disease is primarily related to chronic plaque accumulation in a susceptible host. Major bacterial species implicated in periodontitis are *Porphyromonas gingivalis, Treponema denticola, Tannerella forsythia, Aggregatibacter actinomycetemcomitans* and others^8,12^. *Prevotella intermedia* occurs frequently in the subgingival samples of periodontitis patients^8^. Further, presence of *Prevotella* has been positively correlated with clinical attachment loss^13^, bleeding on probing^14^, and periodontal ()inflammation^15^. *P. intermedia* is a Gram-negative, non-motile, rod-shaped, bacterium that requires strict anaerobic conditions for growth. In addition to oral infections, *P. intermedia* has also been detected from nonoral sites, e.g., NOMA (cancrum oris) lesions^16^ and bacterial tracheitis in children^17^. Further, chronic oral infections such as periodontitis in which *P. intermedia* is a major species, increase the risk of systemic diseases, such as atherosclerosis, pre-term delivery of low birth-weight infants^18,19^. Importantly, *P. intermedia* is known to be resistant to several antibiotics including cephalosporins, penicillins and tetracyclins^20–22^. Since *P. intermedia* is not an exogenous pathogen and is a part of normal oral microbiota, its complete elimination is not possible. Despite the remarkable clinical significance *P. intermedia* has, little is known about the molecular basis for its virulence. For *P. intermedia* to survive in a complex and competitive oral environment, it is imperative that it can adhere to surfaces and integrate into plaque biofilm. In general, bacterial cells dispersed from mature plaque biofilms are collected by saliva and can be regarded as planktonic cells. Such planktonic bacterial cells can reattach to oral surfaces and initiate new biofilm growth. Recent knowledge from the literature suggests that virulence potentials of the biofilms and the planktonic cells are remarkably different^23^. Previously, even though different aspects of *P. intermedia* as part of plaque biofilm have been studied, extracellular proteins secreted by this species (secretome), in biofilm or in planktonic life form, has not been investigated.

## Results

### Analysis of the secretomes of *P. intermedia* biofilm and planktonic cells

**Figure 1:**
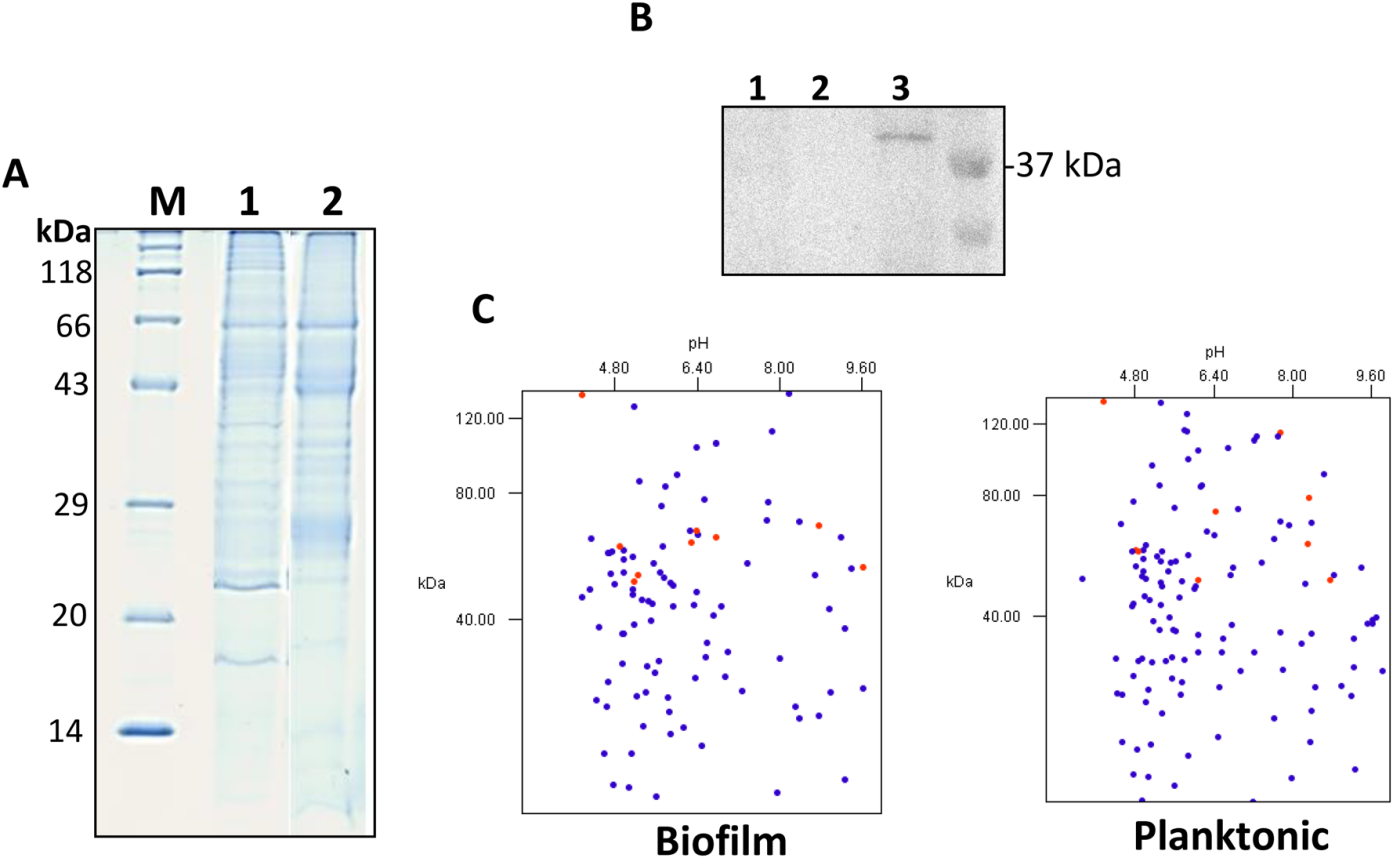
Analysis of the proteome of *P. intermedia* biofilm and planktonic cells. (**A**) SDS–PAGE gel showing protein bands from protein preparations: biofilm (lane 1) and planktonic cells (lane 2). (**B**) Western blot analysis of the secretome preparations (lane 1 = biofilm, lane 2 = planktonic) and the WCP (lane 3) using an antibody for the cytoplasmic marker protein FtsZ. (**C**) Protein sequences from LC–MS analysis of the secretome were analyzed by an in silico 2DE tool.

Protein preparations (Fig. 1) from *P. intermedia* were analyzed by LC–MS/MS. Database search (NCBI-nr) revealed 109 proteins from the biofilm (Suppl. File S1) and 136 proteins from planktonic cells (Suppl. File S2). To ensure that the secretome preparations from the biofilms and the planktonic cells did not contain cytoplasmic proteins due to cell lysis, western blot analysis was performed using an antibody against a cytoplasmic marker protein FtsZ. Panel B in Fig. 1 shows the presence of the marker protein from the whole cell protein preparation from *P. intermedia*, but not from the secretome preparations. As depicted in a theoretical 2DE map of the secretome, the MW of the secreted proteins ranged between 5 and 130 kDa (Fig. 1C). In both biofilm and planktonic cells, with respect to predicted pI values, majority of the proteins formed a cluster with the pI range of 4.0 and 6.5.

**Figure 2:**
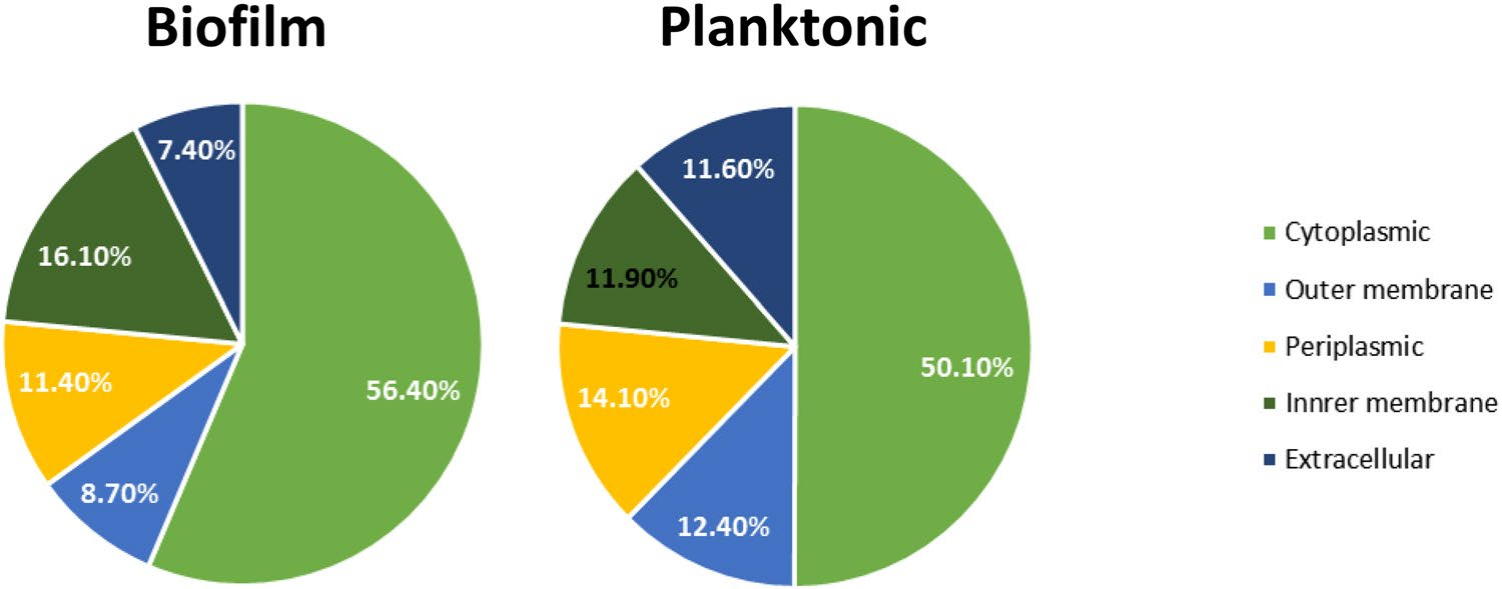
Subcellular localization of proteins. Protein FASTA sequences were analyzed for their subcellular localization using the bioinformatics tool CELLO2GO. The results obtained were compared with other predictions tools such as PSORTb and SignalP.

Protein sequences of the secretomes of *P. intermedia* biofilms and planktonic cells were analyzed for the route of their extracellular release by various bioinformatics tools. Biofilm and planktonic preparations showed 17 and 33 signal peptide-bearing, 13 and 18 lipobox bearing and 2 and 0 TatP signal bearing proteins respectively. Transmembrane alpha helices were found in 4 and 6 proteins from biofilm and planktonic preparations. Subcellular localization analysis (Fig. 2) revealed that the secretome from planktonic cells contained more extracellular proteins (11.6%) than the biofilms (7.4%). Proteins of cytoplasmic origin were more in the biofilm (56.4%) than the planktonic cells (50%). Further, 40–50% of the proteins identified from the secretomes were unique to biofilm or planktonic cells. Importantly, proteins such as superoxide reductase, sensor histidine kinase, C40 family peptidase, elongation factor Tu, threonine synthase etc. were unique to biofilm secretome and were not detected in the planktonic secretome.

### Potential virulence proteins in *P. intermedia* secretome

Virulence potential of the *P. intermedia* was assessed by in silico prediction of virulence factors using the online tools “VirulentPred” and “VFDB” (Virulence Factor DataBase). We found that 31 proteins from the biofilm secretome and 30 proteins from planktonic secretome were predicted to be virulent (Tables 1 and 2). Hemin-binding protein, porin family protein, OmpA, thioredoxin, Omp-28, tetratricopeptide-binding protein molecular chaperones DnaK and GroES were some of the major proteins that were predicted to possess virulence potential.

**Table 1:** Proteins with predicted virulence properties from *P. intermedia* biofilm.

Reference number	Protein
WP_014710387.1	DNA starvation/stationary phase protection protein
WP_028905462.1	Peptidase M6
WP_028905527.1	Sensor histidine kinase
WP_028905084.1	Tetratricopeptide repeat protein
WP_028905748.1	Hemin-binding protein
WP_014710403.1	Thiol reductase thioredoxin
WP_004356500.1	Xaa-Pro aminopeptidase
WP_004364886.1	Trypsin
WP_028905224.1	Peptidyl-prolyl cis–trans isomerase
WP_013265509.1	NADH oxidase
WP_028904949.1	Peptide ABC transporter substrate-binding protein
WP_018667829.1	ATPase
WP_015531502.1	MFS transporter
WP_172460529.1	ROK family protein
WP_028904772.1	Urocanate hydratase
WP_028904668.1	Ribulose-phosphate 3-epimerase
WP_007835729.1	Asp/Glu/hydantoin racemase
WP_005332057.1	N-acetylmuramoyl-L-alanine amidase
WP_007133390.1	Molecular chaperone DnaJ
MBP5257375.1	Acetyltransferase
WP_028906355.1	Substrate-binding domain-containing protein
WP_028904901.1	Energy transducer TonB
WP_007411110.1	Anthranilate phosphoribosyltransferase
WP_028906371.1	1-Acyl-sn-glycerol-3-phosphate acyltransferase
MBO5313912.1	Membrane protein M15
WP_028905881.1	Multidrug ABC transporter ATP-binding protein
MBF1618150.1	Type IV secretion protein Rhs
WP_028906306.1	l-asparaginase
WP_014709317.1	YkgB family protein
MBQ7451373.1	Threonine synthase
MBP3838531.1	HAD-IA family hydrolase

**Table 2:** Proteins with predicted virulence properties from *P. intermedia* planktonic cells.

Reference number	Protein
WP_014710403.1|	Thiol reductase thioredoxin
WP_028905059.1|	Trypsin
WP_025000944.1|	DNA starvation/stationary phase protection protein
WP_014709654.1|	Peptidase M6
WP_028906361.1|	DNA topoisomerase II
WP_028905748.1|	Hemin-binding protein
WP_028905169.1|	DUF4595 domain-containing protein
WP_014709619.1|	Enoyl-ACP reductase
WP_028905189.1|	PorT family protein
MBR7087708.1	Amino acid adenylation domain-containing protein
MBP7359878.1	Chemotaxis protein
MBO7539992.1	DNA polymerase III subunit gamma/tau
MBQ3767790.1	ATP-binding protein
WP_014709212.1|	Peptidylprolyl isomerase
MBO7578384.1	TolC family protein
WP_014709366.1	ABC transporter
MBP8758149.1	DUF1622 domain-containing protein
MBR2882634.1	Ankyrin repeat domain-containing protein
WP_099836288.1	DNA-binding response regulator
WP_097549978.1	tRNA epoxyqueuosine(34) reductase QueG
MBA7488061.1	Calcineurin-like phosphoesterase C-terminal domain-containing protein
MBP5424796.1	Sigma-70 family RNA polymerase sigma factor
WP_100190220.1	Peptidase M20
WP_100356678.1	Type IV secretion protein Rhs
WP_088437864.1	ABC transporter ATP-binding protein
WP_099984831.1	Glycosyltransferase
MBP9983829.1	MarR family transcriptional regulator
WP_099976545.1	HAMP domain-containing histidine kinase
RKW57308.1	SDR family oxidoreductase
WP_097656281.1	TonB-dependent receptor

### Gene Ontology analysis

**Figure 3:**
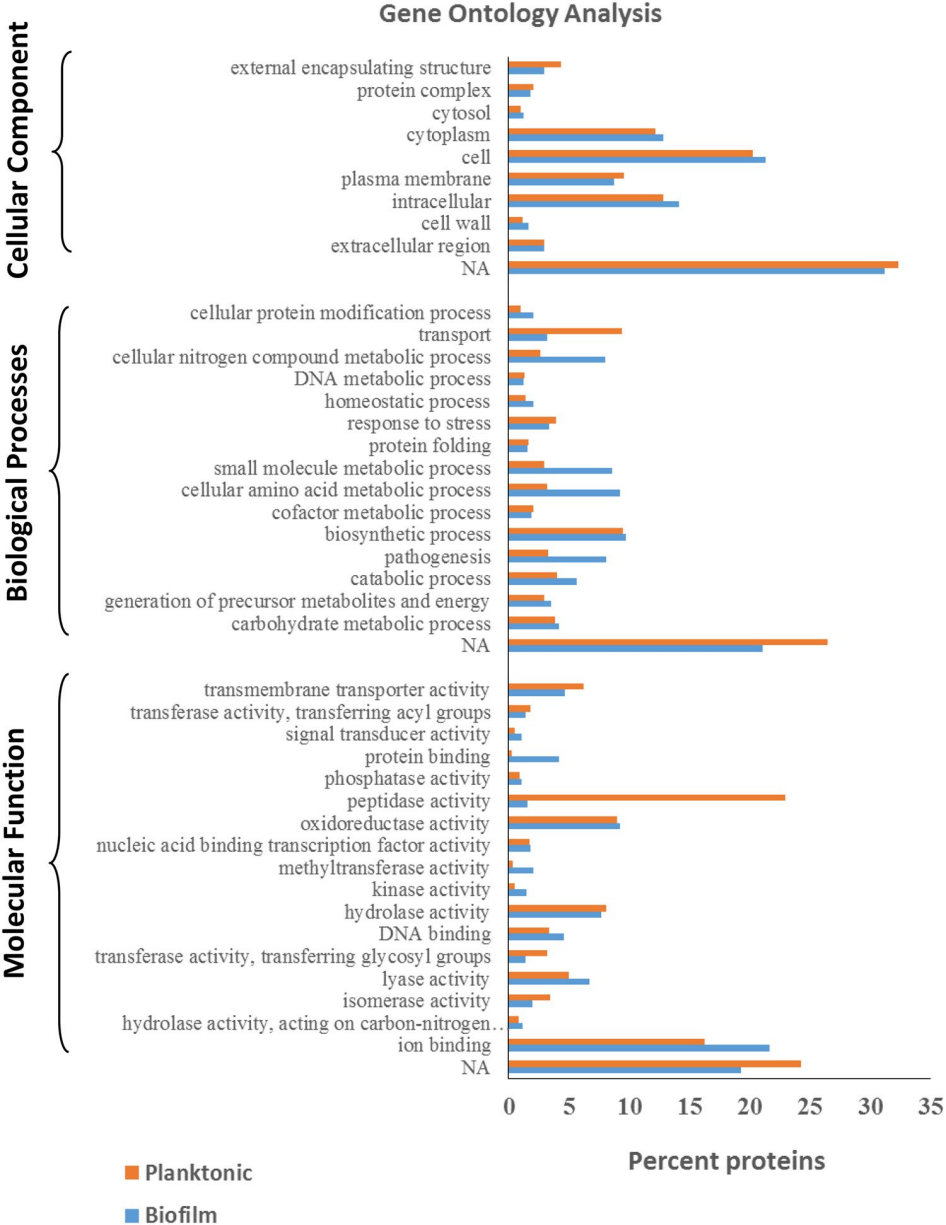
Gene Ontology analysis of *P. intermedia* proteomes from biofilm and planktonic cells. Gene Ontology annotation was achieved using Blast2GO and an online software “CELLO2GO”. Protein sequences were grouped into 3 categories based on their properties and functions.

Gene Ontology (GO) analysis of the amino acid FASTA sequences of the *P. intermedia* secretomes was achieved by using the tools Blast2GO and CELLO2GO. As shown in Fig. 3,marked differences in the percentage of proteins was found with GO annotations in “biological processes” and “molecular functions”. In the category biological processes, biofilm secretome showed higher number of proteins in the case of catabolic process, pathogenesis, cellular amino acid metabolic process, small molecule metabolic process, cellular nitrogen compound metabolic process, while proteins in the “transport” group were higher in planktonic secretome. In the category “molecular function”, biofilm showed higher proteins with “protein binding”, “methyltransferase” and “kinase” activities. The planktonic secretome showed higher number of proteins with peptidase activity.

### Functional protein association network analysis

**Figure 4:**
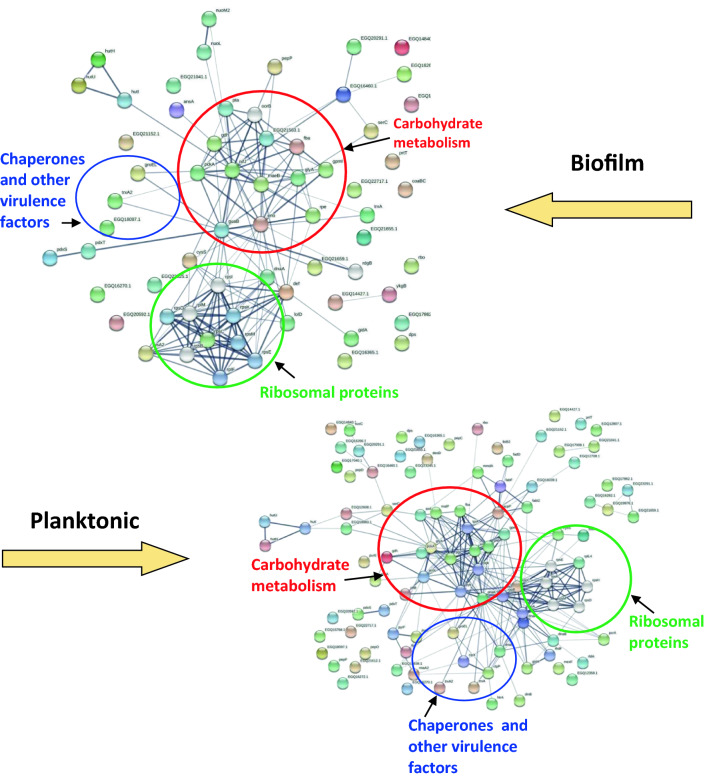
Functional protein association networks of *P. intermedia* proteome from biofilm and planktonic cells. The online tool STRING was used for grouping the secreted proteins based on functional networks. Minimum interaction scores were set at a strong confidence level of 0.7. The three major network groups formed are shown in dotted circles. The thickness of the lines in the network indicates the strength of data support.

As seen in Fig. 4, *P. intermedia* secretome proteins formed three major groups in the STRING network, i.e., carbohydrate metabolism, ribosomal proteins, and chaperones/virulence proteins. Components of the sugar metabolism network were glutamate dehydrogenase, glucose-6-phosphate isomerase, phosphoenolpyruvate carboxykinase, fructose-1,6-bisphosphate aldolase, serine hydroxymethyltransferase, and 2,3-bisphosphoglycerate-independent phosphoglycerate mutase.

Putative virulence-associated proteins and molecular chaperones such as thioredoxin, DnaK, Dps, and GroEL formed another cluster. The ribosomal protein cluster included RplF, RpsE, RpsM, RpsB rSA2, and RplL5 in both biofilm and planktonic cells (Fig. 4).

### Inflammatory potential of the *P. intermedia* secretomes

**Figure 5:**
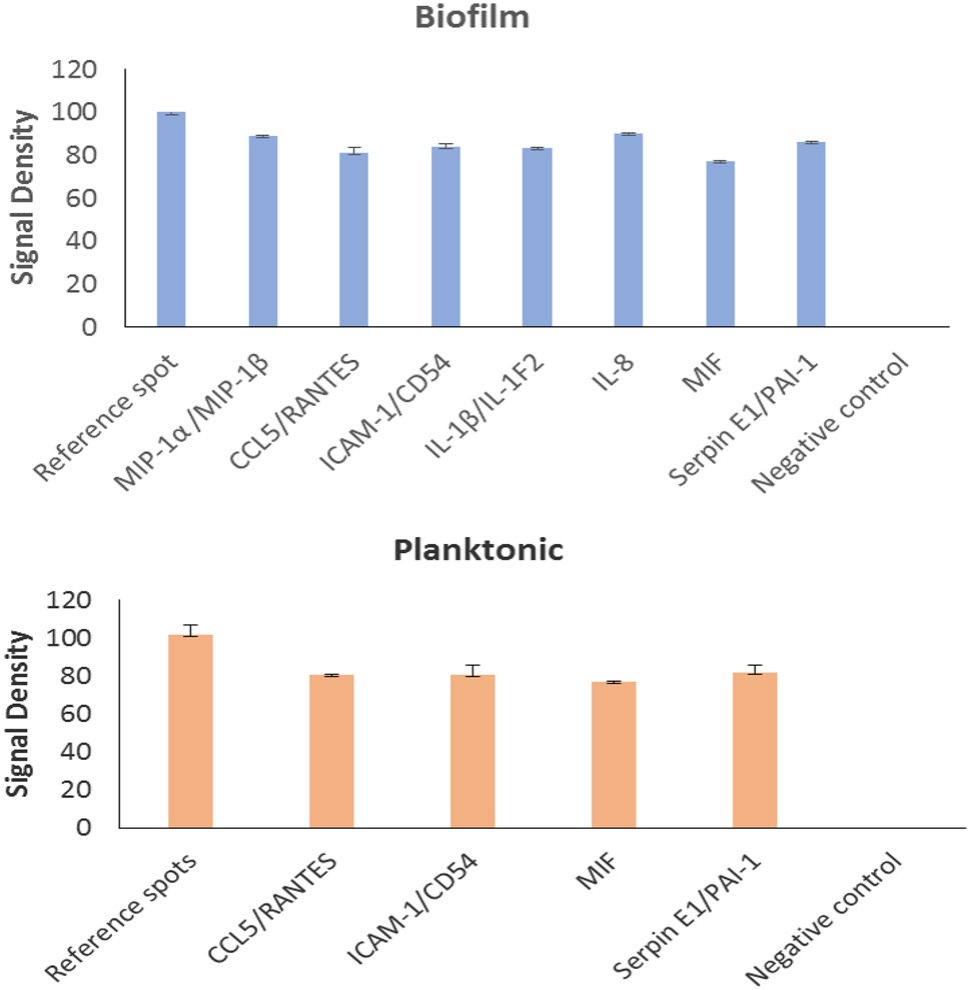
Cytokine induction from human whole blood by *P. intermedia* biofilm and planktonic cells. Human whole blood collected from a healthy volunteer was stimulated by secretome preparations from the biofilms and planktonic cultures of *P. intermedia* for 24 h. The cytokines produced were detected by using Proteome Profiler™ membrane array. Means (SD) of signal densities of spots were determined using Gene Tools analysis software in Syngene Imaging System.

To get a preliminary insight into the inflammatory potential, human whole blood was stimulated with *P. intermedia* secretomes from biofilm and planktonic cultures. As determined by signal densities of cytokine spots on a membrane array (Fig. 5), secretome preparations from both biofilm and planktonic cells induced similar levels of CCL5/RANTES, ICAM-1, MIF, and Serpin E1. The biofilm secretome additionally induced cytokines MIP-1α/MIP-1β, IL-1β, and IL-8.

## Discussion

Protein secretion is a fundamental physiological process among bacteria^24,25^. However, the components of the secretome may vary depending on the niche where the bacteria are located. Biofilm life is a modus operandi for most oral bacteria^9^. Bacterial residents of plaque biofilms continuously release cellular components into the extracellular environment in the biofilm^26^. Interestingly, in this study, proteins such as superoxide reductase, sensor histidine kinase, C40 family peptidase, elongation factor Tu, threonine synthase, which are known to play important roles in bacterial virulence and colonization in the host, were unique to biofilm secretome. Thus, fundamental differences, including those with respect to protein secretion, may exist between the biofilm and planktonic mode of life.

The possibility of contamination of the secretome preparations with subcellular proteins resulting from cell lysis was ruled out by using an established cytoplasmic marker protein Fts-Z^27^ which was undetected in all *P. intermedia* secretome preparations.

Potential virulence factors in the secretome preparations were predicted by in silico analysis. While the total number of predicted virulence factors did not differ between the two secretomes, only six proteins, including thioredoxin, trypsin and hemin-binding protein, were common between them. This is an interesting finding given the fundamental differences of bacteria in biofilm and planktonic modes of life. In the subgingival environment where *P. intermedia* is part of the plaque biofilm encountering a multitude of host challenges, it probably needs to secrete an entirely different set of proteins with virulence potential compared to the planktonic form of life. Of noteworthy virulence factors in the biofilm secretome were thioredoxin, hemin-binding protein, peptidase M6, NADH oxidase and trypsin. In the case of planktonic secretome, enoyl-ACP reductase, PorT family protein, peptidase M20, and glycosyltransferase were some of the important ones. Interestingly, histidine kinases, which were found in both biofilm and planktonic secretomes, are recently proposed to be attractive antibacterial drug targets^28^. Further, hemin-binding protein that plays a role in bacterial interaction with host cells^29^, glycosyltransferases that mediate biofilm formation^30^. Similarly, virulence potential of other proteins such as NADH oxidase^31^ and thioredoxin^32^ has been well established in several other bacterial pathogens.

To get an insight into the functional significance of the proteins identified from the *P. intermedia* proteomes, Gene Ontology analysis was carried out on the FASTA sequences. The biofilm secretome contained higher percentage of proteins in “biological processes” category with catabolic process, cellular amino acid metabolic process, cellular nitrogen compound process. Biofilm secretome also showed higher number of proteins with protein binding, methyl transferase and kinase activities. Interestingly, the planktonic secretome appeared to possess higher percentage of proteins with peptidase activity, compared to the biofilm secretome. In another important periodontal pathogen *P. gingivalis*, certain peptidases were important for the survival of the species but not sufficient for its virulence^33^.

The biofilm and planktonic secretomes were analyzed for their functional network associations by using STRING. Three function-based groups were identified, carbohydrate metabolism, ribosomal proteins and a third network comprising of virulence factors and chaperones. Key enzymes in carbohydrate metabolism, glutamate dehydrogenase, glucose-6-phosphate isomerase and phosphoenolpyruvate carboxylase were part of the network. Predicted virulence factors that also function as molecular chaperones, such as thioredoxin, DnaK, dps and GroEL formed a distinct network. Several years ago, the ability of *P. intermedia* DnaK to function as a molecular chaperone was assessed by a luciferase folding assay^34^. In periodontitis, stresses such as temperature and pH increase, increased flow of gingival crevicular fluid, and oxidative stress resulting from phagocytic cells. In response to these stresses, *P. intermedia* may show increased expression of heat-shock proteins such as DnaK. This may contribute to enhanced virulence of the species as well as its survival in stress conditions. In a study that investigated the effect of oxidative stress on *P. intermedia* protein exopression^35^**,** several proteins from the above functional networks, including fructose-1, 6-bisphosphate aldolase, reductases, ribosomal proteins and molecular chaperones like thioredoxin, DnaK were upregulated when bacteria were exposed to molecular oxygen. The significance of the secretion of these proteins may possibly be related to the ability of *P. intermedia* to travel beyond the oral cavity and colonize distant body sites where the species must adapt to oxygenated tissues.

*P. intermedia* has been considered a major periodontal pathogen because of its established role in initiation and development of periodontitis by inducing a variety of proinflammatory cytokines, proteases, and matrix metalloproteinases. Important cytokines such as IL-8, IL1-β, and macrophage inflammatory proteins were induced by the biofilm secretome. *P. intermedia* and its components have been shown to induce IL-8, IL1-β, TNF-α and MMPs^36,37^. The role of these and other proinflammatory cytokines such as MIP-α and MIP-β in periodontitis has been well established^38^. Importantly, certain bacteria-secreted proteins stimulate cytokines only in their secreted-form, not when they are within the bacterial cell^39^. This type of specificity may suggest possible roles of *P. intermedia* secretome proteins beyond the oral cavity.

In conclusion, the current proteomics data on the biofilm- and planktonic- secretomes may provide new insights into virulence mechanisms of *P. intermedia*. For example, the role/potential of the predicted virulence factors in the secretomes may be studied by overexpressing the respective genes in a suitable system, and by constructing specific knockout mutants for further studies using in vitro and/or in vivo models.

## Methods

### Biofilm and planktonic cultures

*P. intermedia* ATCC 25,611 was grown on brucella blood agar containing 5% sheep blood in anaerobic atmosphere (10% H_2_, 5% CO_2_, 85% N_2_) at 37 °C using Anoxomat™ Mark II anaerobic gas filling system (Mart Microbiology, The Netherlands) in jars for 3 days. Biofilms and planktonic cultures were grown as described earlier with some modifications^40^. Bacterial colonies were harvested from agar plates with sterile disposable loops and suspended in brucella broth. The bacterial cells were washed at least once by suspending in brucella broth and then collected by centrifugation at 5000×*g* for 5 min. The washed bacterial cell pellet was resuspended in 1 ml brucella broth to make a stock suspension. A final bacterial suspension of OD_600_ = 1 was prepared after measuring the optical density of the stock suspension.

Biofilms and broth cultures for planktonic bacterial growth were initiated by inoculating 24-well plates and microfuge tubes, respectively, containing 900 µl brucella broth with a 100-µl aliquot from an OD_600_ = 1 suspension of each species. Wells (for biofilms) or tubes (for planktonic cells) with only broth were considered as negative control. The plates/tubes were incubated in the same culture conditions as above for 24 h. At the end of incubation period, supernatant broth from biofilms was aspirated and the supernatants from planktonic cultures were collected by centrifugation at 5000 × g for 10 min. To eliminate the chances of the presence of any intact bacterial cells, the supernatants were filtered through a 0.22 µm sterile syringe filter (Millipore, Germany). These supernatants were subjected to secretome preparation.

### Preparation of secretome

The secretomes were prepared by extracting proteins using tri-chloroacetic acid (TCA) precipitation method as described previously with modifications^41,42^. TCA stock (100% w/v) was mixed with supernatant culture broth at 1:4 ratio and incubated for 30 min at − 20 °C. After centrifugation at 14,000×*g* for 20 min at 4 °C, traces of acid in the pellet were removed by washing twice with 0.5 ml cold acetone, followed by complete air-drying in a fume hood. The samples were desalted by ultrafiltration through 3 K Ultra-0.5 centrifugal filter devices (Amicon) at 14,000×*g* for 15 min at 4 °C. After discarding the flow-through, concentrates in the columns were finally eluted from columns by centrifugation at 1000×*g* for 2 min at 4 °C. Broth without bacteria was incubated in parallel and used as negative control.

### Whole cell protein preparation

*P. intermedia* colonies harvested from brucella blood agar plates were washed in sterile PBS by centrifugation at 5000×*g* for 5 min. The pellet was resuspended in lysis buffer containing 1 mg/ml lysozyme and 1 mM phenyl methyl sulfonyl fluoride (PMSF) and incubated for 4 h at 4–8 °C. The samples were subject to sonication in Omni-Ruptor 4000 (Omni International, USA) at a pulse rate 40 for 8 times (1 min sonication with 1 min interval on ice). The lysates were centrifuged at 10,000×*g* for 10 min at 4 °C.

### Determination of protein concentration

Protein concentrations in secretome preparations and whole cell lysates were estimated by Quick Start™ Bradford protein microplate standard assay (Bio-Rad, USA) as per manufacturer instructions.

### SDS–PAGE

Protein samples were mixed with 5 × Laemmli sample buffer (125 mM tris, pH 6.8; 6% glycerol, 2% SDS; 5% beta-mercapthoethanol; 0.025% bromophenol blue) and boiled at 95 °C for 5 min. The samples were loaded on a 15% SDS-PAGE gel [4% stacking gel (4% acrylamide; 68 mM tris, pH 6.8; 0.2% SDS), 15% separating gel; 375 mM tris, pH 8.8; 0.1% SDS]. Electrophoresis was run at 150 V for 75 min (Mini-protein II Dual Slab Cell, Bio Rad, USA) and the protein bands were visualized using Coomassie blue.

### Western blot analysis

To ensure that the secretome preparations did not contain proteins that originated due to cell lysis, western blot analysis of whole cell lysate and secretome preparations was performed. Protein bands on the gel were transferred onto a PVDF membrane using Trans-Blot® Turbo™ transfer system (Bio-Rad). To avoid unspecific binding, membrane was blocked with 5% skimmed milk overnight at 4 °C. As primary antibody, an antibody against the cytoplasmic marker protein, Ftsz (Agrisera AB, Sweden) was used at 1:1000 dilution and incubated 1 h at room temperature. The membrane was then incubated as above with a peroxidase conjugated goat antirabbit secondary antibody (1:5000). The membrane was washed with tris-buffer saline containing Tween-20 (TBST). Finally, the bound antibodies on the membrane were detected by using SuperSignal™ West Pico chemiluminescence substrate (Pierce, USA) and images were acquired in G:Box Imaging System (Syngene, UK).

### Identification of proteins by nano-LC–ESI–MS/MS

For the identification of proteins in the secretomes from biofilms and planktonic cultures, mass spectrometry was performed at Proteome Factory (Proteome Factory AG, Berlin, Germany) using nano-liquid chromatography-electrospray ionization-tandem mass spectrometry (nano-LC–ESI–MS/MS). With an Agilent 1100 nanoHPLC system (Agilent, Waldbronn, Germany) interfaced to an Orbitrap Velos (Thermo Scientific, Bremen, Germany) via a nanoelectrospray ion source. After pooling replicate samples from EVs preparations, proteins were reduced, alkylated and digested by trypsin (Promega, Mannheim, Germany). Then, 400 ng of the resulting peptides were subjected to the nanoLC–ESI–MS/MS. 1% acetonitrile/0.5% formic acid was used as eluent for 5 min to trap and desalt the peptides on the enrichment column (Zorbax 300SB-C18, 0.3 × 5 mm, Agilent). A water/acetonitrile (both supplemented with 0.1% formic acid) gradient from 5 to 40% acetonitrile was then used within 120 min to separate the peptides on a Zorbax 300SB-C18, 75 µm × 150 mm column (Agilent). The mass spectrometer automatically recorded mass spectra, and tandem mass spectra were data-dependently acquired for multiply charged ions. Protein identification was made using the Mascot search engine (Matrix Science, London, England) against the bacterial subset of the RefSeq protein database (National Center for Biotechnology Information), and a database with common protein contaminants. For MS/MS spectra where assignment of the precursor ion’s charge state was missing, search parameters for ions from ESI–MS/MS data acquisition was set to "2 + , 3 + or 4 + " according to the instrument's and method's standard charge state distribution. The search parameters were: Fixed modifications: Carbamidomethyl (C); variable modifications: Deamidated (NQ), Oxidation (M); Peptide Mass Tolerance: ± 3 ppm; Fragment Mass Tolerance: ± 0.6 Da; Missed Cleavages: 2. The inclusion criterion was: peptides that match with a score of 20 or above. Mass spectrometry data, with the project acession number PXD029419, has been deposited at PRIDE archive (https://www.ebi.ac.uk/pride/archive/) repository. The data files can be accessed with the username reviewer_pxd029419@ebi.ac.uk and the password gtYGw1hi.

### Bioinformatics analysis

Most of the bioinformatics analyses were performed as described previously with some modifications^43^. Theoretical in silico (2-DE) image of the proteins was acquired by using the tool JVirGel, version 2.0 (http://www.jvirgel.de/index.html)^44^. The subcellular localization of the secretome proteins was predicted using the PSORTb tool, version 3.0.2 (https://www.psort.org/psortb/)^45^. Signal-peptide bearing proteins were predicted by using the online tool SignalP, version 5.0 (http://www.cbs.dtu.dk/services/SignalP/abstract.php)^46^. Lipoproteins in the secretomes were predicted using the prediction tools LipoP (http://www.cbs.dtu.dk/services/LipoP/) and PRED-LIPO (http://bioinformatics.biol.uoa.gr/PRED-LIPO/input.jsp)^47^. Further, the prediction tool TatP (http://www.cbs.dtu.dk/services/TatP/), was used to predict proteins secreted via the Twin-arginine translocation pathway (Tat-pathway)^48^.

### Function prediction analysis

Gene Ontology (GO) of the proteins was analyzed by using the amino acid FASTA sequences. For this, GO annotations were analyzed and plotted using the tools OmicsBox version 1.3.11 (https://www.biobam.com/download-omicsbox/)^49^, and CELLO2GO^50^. Functional association networks within the secretome proteins were determined using the tool STRING (https://string-db.org/)^51^. Minimum interaction scores were set at a strong confidence level of 0.7.

### Prediction of virulence factors in the EVs proteomes

VirulentPred (http://203.92.44.117/virulent/) was utilized to predict potent virulence factors in the secretomes^52^, along with the Virulence Factor Data Base (VFDB; http://www.mgc.ac.cn/VFs/).

### Stimulation of human whole blood with secretome protein preparations

Whole blood collected from a healthy human volunteer was stimulated with *P. intermedia* biofilm and planktonic secretome preparations for 24 h. In a 24-well plate, whole blood was added to the wells and stimulated with 20-µg of secretome protein preparations. After incubating at 37 °C and in 5% CO_2_ in air for 24 h, plasma was separated by centrifugation at 500×*g* for 3 min and used for cytokine profiling. Duplicate wells containing whole blood but treated with 20 µl sterile PBS were used as negative control.

### Cytokine detection using Proteome Profiler™ arrays

Plasma separated from human whole blood stimulated with secretome preparations was applied onto cytokine array membranes for cytokine detection as follows: Nitrocellulose membrane with 36 different capture antibodies spotted in duplicate was used to determine the relative levels of cytokines. Unspecific binding was blocked with assay buffer for 1 h at room temperature. The secretome-stimulated PBMC sample (1.5 ml) was diluted in assay buffer with 15 µl of reconstituted human cytokine array detection antibody cocktail and incubated at room temperature for 1 h. After washing, the array was treated with streptavidin HRP for 30 min at room temperature on a rocking platform shaker. The array was finally incubated with chemiluminescence reagent for 10 min and images were acquired in Syngene G:Box Imaging System. The positive signals visualized on the array were identified by comparing with the transparency overlay template with the pairs of reference spots in three corners of each array. Mean spot pixel densities were calculated from duplicate spots by analyzing the image using the software provided with G:Box Imaging System.

### Ethical approval

This study was approved by the ethical committee of the Health Sciences Center, Kuwait University (DR/EC/3413), and has been carried out in full accordance with the World Medical Association Declaration of Helsinki. The blood donor received written information about the nature and purposes of the study and a written informed consent was obtained upon the volunteer’s approval to participate.

## Supplementary Information

Supplementary Information 1.

Supplementary Information 2.

Supplementary Information 3.
