# Supplementary material for: Proteomic analysis of the periodontal pathogen Prevotella intermedia secretomes in biofilm and planktonic lifestyles
Source: Sci Rep. 2022 Apr 4;12:5636. doi: 10.1038/s41598-022-09085-0 (PMC8980031; doi:10.1038/s41598-022-09085-0)
Supplement: Supplementary file 1 — Supplementary Information 1. [file 41598_2022_9085_MOESM1_ESM.pdf]

Proteomic analysis of the periodontal pathogen *Prevotella intermedia* secretomes in biofilm and planktonic lifestyles

Project: 20L144_Kuwait

Preparation:

- Lyophilisated sample´s were diluted in 50ul 8M Urea
- Bradford determination
- 5ug per sample is loaded on 15% SDS-Polyacrylamid-Gel Run conditions: 150V, 400mA max., 75min

15%SDS-PAGE:

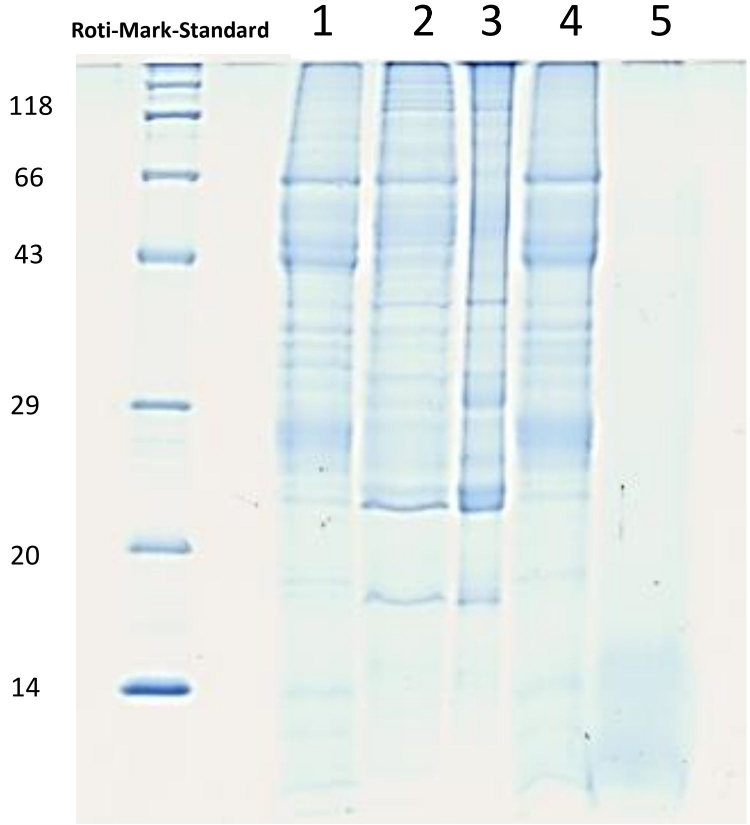

Fig 1A: Original gel picture used in Fig 1A in the manuscript. Only the lanes 2 and 4 from the above gel are used in Fig 1A in the revised manuscript (labelled as lanes 1 and 2).

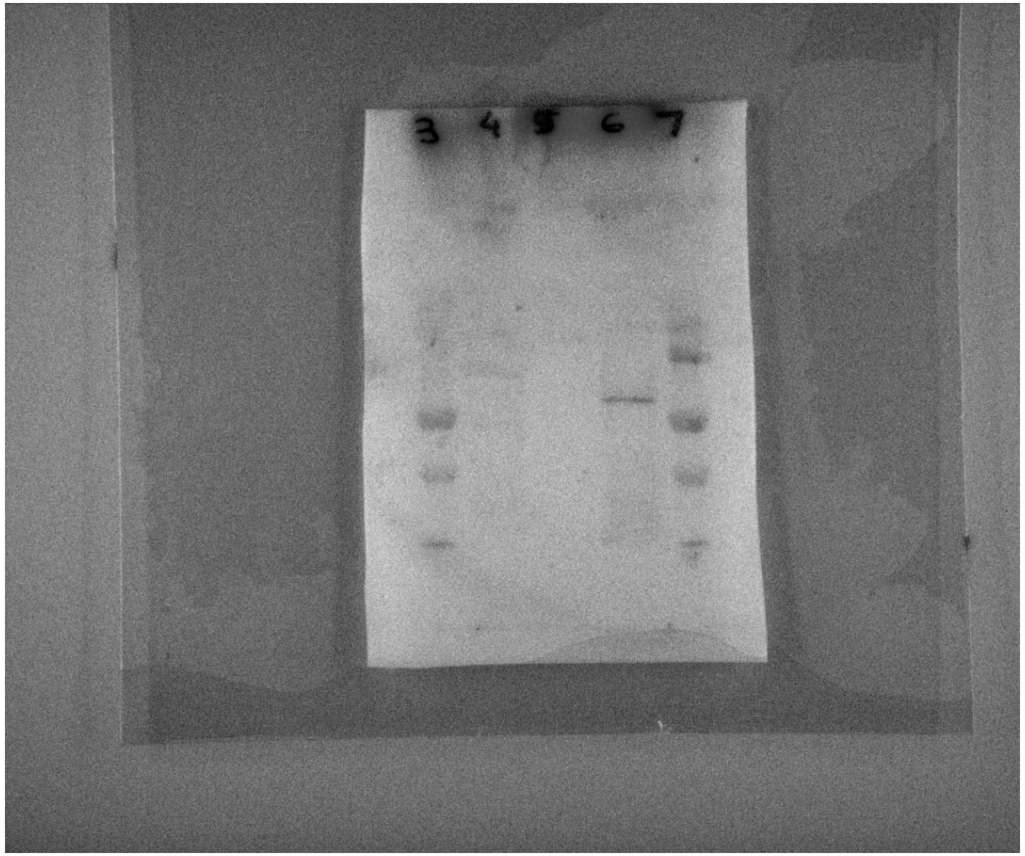

Fig 1B: Original blot used in Fig 1B in the manuscript.
